# A review about methods for supporting failure risks analysis in eco-assessment

**DOI:** 10.1007/s10661-021-09175-y

**Published:** 2021-06-23

**Authors:** Christian Spreafico

**Affiliations:** Department of Management, Information and Production Engineering, University of Bergamo, Via Marconi 5, Dalmine, 24044 Bergamo, Italy

**Keywords:** Life cycle assessment (LCA), FMEA, Failure determination, Risk analysis

## Abstract

This paper critically reviewed 106 scientific papers proposing methods to enrich eco-assessment with failure determination and risk assessment. The provided research perspective is new and significantly different from the reviews in the literature which are mostly limited to analyse the environmental impacts of uncertainties and off-design functioning rather than the failures. The analysis, based on the contributions of the literature over more than 20 years, was carried out manually and allowed to identify and classify the application fields, the types of identifiable failures and the approaches used for their determination, for the analysis of their risk of occurrence and for their eco-assessment. The different classifications have also been intersected with each other and all the proposed approaches have been discussed in detail, highlighting the advantages and disadvantages in relation to eco-assessment. From the study emerged a growing and heterogeneous interest on the subject by the scientific community, and a certain independence of the analysed methods with respect to traditional approaches of both failure risk analysis and eco-assessment. Great attention of the methods about product functioning has been highlighted, in addition to the use of tests, simulations, FMEA (failure mode and effect analysis)-based approaches and knowledge databases to determine the failures, while statistical methods are preferred to support risks analysis and LCA (life cycle assessment) for environmental impact calculation. If, in the coming years, this argument also spreads in industry, the results provided by this review could be exploited as a first framework for practitioners.

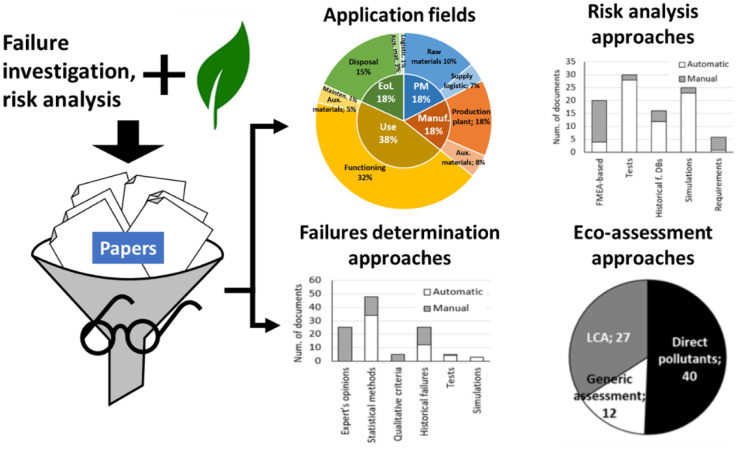

## Introduction

In recent years, the perception of environmental problems in industry has increased considerably, and today, there are many ways to evaluate the sustainability of a product. In order to objectify this activity, structured methodologies and standardized indicators of the environmental impacts have been introduced.

However, although the most diffused approaches, e.g. life cycle assessment (LCA), have been appreciated for their reliability and completeness in several application fields, both in commercial certifications and in scientific publications (e.g. Spreafico & Russo, 2020; Spreafico, 2021a, b), their main limitation consists in contemplating only standard scenarios, omitting the even important anomalies (Heijungs & Huijbregts, 2004). In fact, the reference standards (e.g. ISO 14040: 2006, ISO, 2006a) establish that the obtained results should be discussed according to the sensitivity analysis, which considers only the uncertainties relating to the assumptions in the inventory and in the allocation of resources, without considering failures, which, on the other hand, can have a much greater influence on impacts (Sugiyama et al., 2005).

The consideration of the failures during the eco-assessment is also useful to improve the reliability of the same analysis, avoiding simplistic assumptions that sometimes one is forced to make or to rely too much on product category rule (PCR) parameters that are not as representative as well as an accurate and ad hoc investigation of the single product (Naderi et al., 2020). In addition, the analysis of product failures and their related risks, during the eco-assessment, can also benefit decision-making about the eco-improvement choices (Matthews et al., 2002).

Those who choose to take this path, to understand on which approaches to address, can consider some reviews from the literature.

The extensive review on failure mode and effect analysis (FMEA) and risk assessment (e.g. Sharma & Srivastava, 2018; Spreafico et al., 2017; Sutrisno & Lee, 2011) can be a starting point for starting the investigation since they provide a broad perspective on the topic of failure determination, comparing a large number of contributions and extrapolating trends and general considerations. However, they provide very marginal information regarding the link with eco-assessment, limited to no more than five relevant contributions without discussing them.

The same problem is also found in the general reviews on eco-assessment (e.g. Bamber et al., 2020; Rossi et al., 2016) which, by comparing and classifying many documents and maintaining an investigation perspective on many issues, cannot provide a detailed view on the subject of our study, or even ignore it.

A more privileged description on the subject could come emerge from those reviews comparing failure determination and eco-assessment. Kania et al. (2014) show us how FMEA is used in this area, discussing its main variations and explaining how its constitutive parts have been adapted. This analysis is useful to understand how to apply the FMEA for environmental problems, but it does not explain how to deal with failures during the assessment of environmental impacts. Puglieri and Ometto (2011) provide a more detailed perspective, comparing how different approaches perturb product requirements to determine possible environmental failures, although without specifying, even in this case, the criteria for eco-assessment. While the review of Igos et al. (2019) can be useful to increase the knowledge of LCA practitioners related to uncertainty and facilitate the application of treatment techniques, many of which can also be exploited to deal with failures. However, the focus of this study is mainly aimed at methodological limitations which, unlike failures, have a limited effect on the overall environmental impact of the product.

Consequently, at today, a review that can shed light on how many and which methods exist to estimate the environmental impacts deriving from product failures is still missing.

The objective of this study to overcome these limitations is to propose a review of all the methods, gathered from scientific publications, that combine failure determination and risk analysis with the assessment of the product’s environmental impacts from cradle to grave.

Its more evident novelties, in addition to the treated objective, concern the following: the broadening of the survey perspective, considering a greater number of documents from the literature compared to previous reviews, and a structured and in-depth analysis approach for all the different constituent sub-topics of investigation aiming to answer the following research questions.

RQ 1: What are the product failures occurring during its life cycle that are generally included in the eco-assessment, and in what way and with what approaches the previous studies from the literature determined them?

RQ 2: How the risk of occurrence of such failures was assessed in the literature and according to which approaches?

RQ 3: With which approaches and in what way the environmental impacts deriving from the determined features have been assessed, and what was the role that the risk of occurrence during this phase?

This study intends to answer each question by presenting all the pertinent approaches identified within the reference pool, classifying them according to methodological and applicative criteria, and adequately discussing the advantages and disadvantages, of all the main approaches, in relation to eco-assessment.

The ultimate goal of the review is to provide practitioners and researchers with a reference framework on the subject, which invites them to improve their environmental analyses by also taking into account failures and, at the same time, suggesting the more suitable approaches to adopt according to the analysed product and the specific environmental issues.

## Research methodology

In this review, all the documents were collected (“Document collection”) and analysed (“Document analysis”) through a multi-step procedure based on an extensive manually supervised automatic review.

### Document collection

During the collection of the sources, we mainly focused on scientific papers rather than other sources since they assure a more scientific content and can guarantee a rigorous revision, and the selection of scientific journals was based on the editor’s notoriety, journals maturity and impact factor, matching to the scope of the journal. For what concern the conferences we selected only the international ones, explicitly dedicated to this topic and with long-lasting and adequate referee processes.

The documents have been searched within Scopus, Google Scholar, Web of Science, ASME digital collection and ProQuest. The used query for Scopus database, within the title, abstract and keywords of the documents, was the following: (FMEA OR FMECA OR EFMEA OR AFD OR (risk AND (assess + OR evaluat + OR analysis)) or failure +) and (((eco + OR green OR environment + OR “life cycle” OR impact +) AND (assess + OR evaluat + OR calculat + OR estim +)) OR LCA). The same query used in Google Scholar returned only 2% of the results provided by Scopus (i.e. 17,000 documents vs 683,296). Then, in order not to lose documents, in Google Scholar, the search was carried out using only the Boolean operators, which according to the guidelines of Google Scholar work. While the truncations and elisions have been omitted and the resulting terms have all been reported and linked by the OR operator, e.g. assessment OR assessments OR assessing). In this way, many more documents were identified and then analysed. The same procedure was also used for the other databases that do not allow a syntactic search in the Scopus mode.

This query was identified following numerous tests, with the aim of favouring recall rather than precision, leaving the selection of documents to a greater extent to the purely manual analysis of titles and abstracts of the articles. This last step, albeit onerous, was necessary due to the breadth and heterogeneity of the jargon used by the relevant documents, which we were able to detect during these tests.

During this activity, various selection criteria were adopted in order to exclude from the pool all the contributions attributable to at least one of these categories: (1) environmental assessments not related to the impacts but to the working environment of the product; (2) analysis of the risks associated to the consequences of the environmental impacts, detected under standard conditions, or the risk analysis is conducted downstream instead of upstream compared to eco-design; and (3) lexical misunderstandings, where the concept of risk is used as a synonym for environmental impact.

The resulting pool of documents counts 106 documents, of which 76 articles from international journals, 27 conference papers and 3 book chapters, spanning from 1997 to today.

**Fig. 1:**
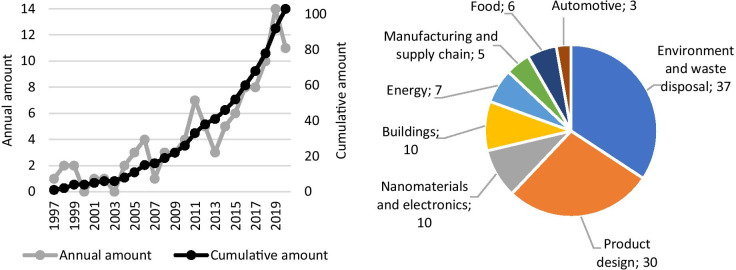
(left) Time distribution (number of publications) and (right) fields of application of the collected methods

The graph represented in Fig. 1 (left) distributes these documents according to a temporal axis, Fig. 1 (right) presents the distribution of the application fields of the considered methods, and Table 1 reports the list of scientific journals of the considered articles, classified according to their topics. From these representations, it is easy to see that the number of annual publications on the subject has grown sharply since the last 5 years, that these contributions investigate a large number of application fields, even very different from each other and which have been published in many journals, in turn on different topics. In addition, the slight predilections towards product design and journals on the environmental problems further confirm the heterogeneous interest of the literature on the subject, given the variety of topics of these classes.

**Table 1:** List of scientific journals of the considered articles, classified according to their topics

Scientific journals main topics	No. of contributions	Scientific journals
Environmental issues and waste management	40	PLOS One (8); Science of the Total Environment (2); International Journal of Environmental Research and Public Health (2); Nature Communications; Ecosystem Health and Sustainability (2); Advances in Water Resources; Agriculture, Ecosystems & Environment; Environment & Ecosystem Science; Environmental Earth Sciences; Environmental Geochemistry and Health; Environment international; Environmental Toxicology and Chemistry; Foods; Journal of Contaminant Hydrology; Journal of Hazardous Materials; Journal of Material Cycles and Waste Management; Ocean Engineering; Journal of Soil Science and Plant Nutrition; Nanotoxicology; Waste Management; Water Science and Technology; International Journal of Environmental Research and Public Health; Pathogens; Nature Climate Change; Journal of Energy Resources Technology; Environmental Health Perspectives; Frontiers in Marine Science; Nature Environment and Pollution Technology; Geology, Ecology, and Landscapes; Geospatial health; Parasites & Vectors
Eco-assessment, eco-design and sustainable engineering	11	Sustainability (6); Environmental Science & Technology; International Journal of Sustainable Engineering; Journal of Cleaner Production; The International Journal of Life Cycle Assessment; Calitatea
Structural and information engineering	11	Journal of Mechanical Design (3); Advances in Space Research; Energy and Buildings; Fullerenes, Nanotubes and Carbon Nanostructures; International Journal on Advanced Science Engineering Information Technology; International Journal of Structural Integrity; Kybernetes; Metalurgija; Journal of Composites Science
Failure and risk analysis	8	Journal of Failure Analysis and Prevention (2); Journal of Risk Research; Risk Analysis (2); Human and Ecological Risk Assessment; Communications in Dependability and Quality Management; European Research Studies
Innovation design and management	3	International Journal on Interactive Design and Manufacturing; Engineering Economics; The Grammar of Technology Development

### Document analysis

The analysis of the documents was conducted using an automatic method for data scanning, followed by a manual check of the automatically retrieved results.

First, the full text of all the collected documents was analysed with Sketch Engine, a natural language process tool for the text mining, which is based on syntactic analysis. During this phase, the two main functionalities of these tools were both exploited. Through the topic mapping, an ordered list of the main arguments treated within the considered pool of documents was automatically obtained. In this case, the sorting criteria used by the software are the number of occurrences of the topics within the overall analysed textual corpus.

Then, each of the main topics was entered as “lemma” to automatically determine all the syntactically related terms through the Word Sketch function. Selecting the retrieved relations between the lemma and the other terms, we accessed to the single sentences containing them and automatically provided by the software. Each sentence was manually analysed to evaluate its pertinence with the research theme and to collecting other information to be collected or further used as novel lemmas. For instance, the automatically extracted topic “automatic”, used as lemma, provided the second term “analysis”. In turn, the two terms (“automatic analysis”), still used as multi-word lemma, provided another term, i.e. “of risk”. Then, analysing one of the automatically provided sentence containing all these three terms, i.e. “an automatic analysis of risk”, we learned that a probabilistic index was used to carry out this goal.

Finally, all the retrieved pertinent results were manually classified and discussed in detail. To analyse the failures determined by the considered methods, and answer to RQ 1, they have been collected and classified according to the LCA ontology (see “Types of failure”), while the approaches proposed for their determination have been classified according to the type, the degree of user involvement, the application fields and the types of failures, by discussing in detail their advantages and limitations (see “Exploited approaches”). To comprehend how the risk of occurrence of the failures is assessed (RQ 2), we proceeded in the same way as for failures, classifying the identified approaches and analysing them in detail (see “Risk assessment approaches”). While to evaluate how the eco-assessment is conducted on the hypothesized failures (RQ 3), the identified approaches (for the eco-assessment) were presented, classified and compared to the other obtained results (see “Eco-assessment approaches”). Finally, to highlight the peculiarities of the considered methods, also useful for completing the answers provided to RQ 1 and RQ 2, we compared the obtained results with those of two previous general reviews on FMEA and on risk assessment (see “Discussion of the results”).

In Table 2 in the Appendix, the main classification of all the analysed documents according to the considered criteria of evaluation are reported, in the following section, the results of the classification are presented and discussed in detail.

**Table 2:** Failures identified by the considered methods, classified according to product life cycle phases and addressed items

Product life cycle phases	Addressed items and types of failures	Number of documents
Pre-manufacturing	Raw materials: variations in the composition and microstructure of the materials that make up the product (e.g. van Harmelen et al., 2016)	20
	Supply logistic: delays, accidents, damages of the goods during the transportation (e.g. Djekic et al., 2019)	5
	Sub-total	25
Manufacturing	Production plant: unexpected failures of the production plant, errors in processing, damages to semi-finished products (e.g. Ibrahim & Chassapis, 2017)	20
	Auxiliary materials for manufacturing: degradation of properties and damages in the supply plants (e.g. Djatna & Prasetyo, 2019)	6
	Sub-total	26
Use	Product functioning: breakages, damages (e.g. Naderi et al., 2020) and human misuses of the product (e.g. Norazahar et al., 2018)	61
	Product auxiliary materials (e.g. De Almeida Júnior & Castro, 2015)	4
	Product maintenance: errors in planning and execution of the interventions (e.g. Hennequin et al., 2018)	1
	Sub-total	66
End-of-life	Disposal: guasti negli impianti e perdite di materiale inquinante (e.g. Lazarova et al., 2012)	23
	Auxiliary materials: (e.g. Lemos & Castro, 2018)	2
	Logistic (e.g. Lemos & Castro, 2018)	1
	Sub-total	26
Total		143

## Failure determination

### Types of failure

In this section, we present the types of failures that the analysed methods can identify. To classify the failures, we used the typical ontology of the LCA according to its reference standards (ISO 14040: 2006 (ISO, 2006a) and ISO 14044: 2006 (ISO, 2006b)), since it provides all the elements to treat them in the most suitable and complete way according to our purposes.

The adopted classification is based on two levels: all failures have been grouped according to the items of the LCA to which they refer (e.g. raw materials, supply logistic), which have therefore been grouped into the four phases of the product life cycle (i.e. pre-manufacturing, manufacturing, use and end-of-life).

Table 1 shows all the types of identified failures, classified according to the items and the phases of the product life cycle, with an indication of the number of documents, among those considered, which explicitly declare to support the detection of such failures.

**Fig. 2:**
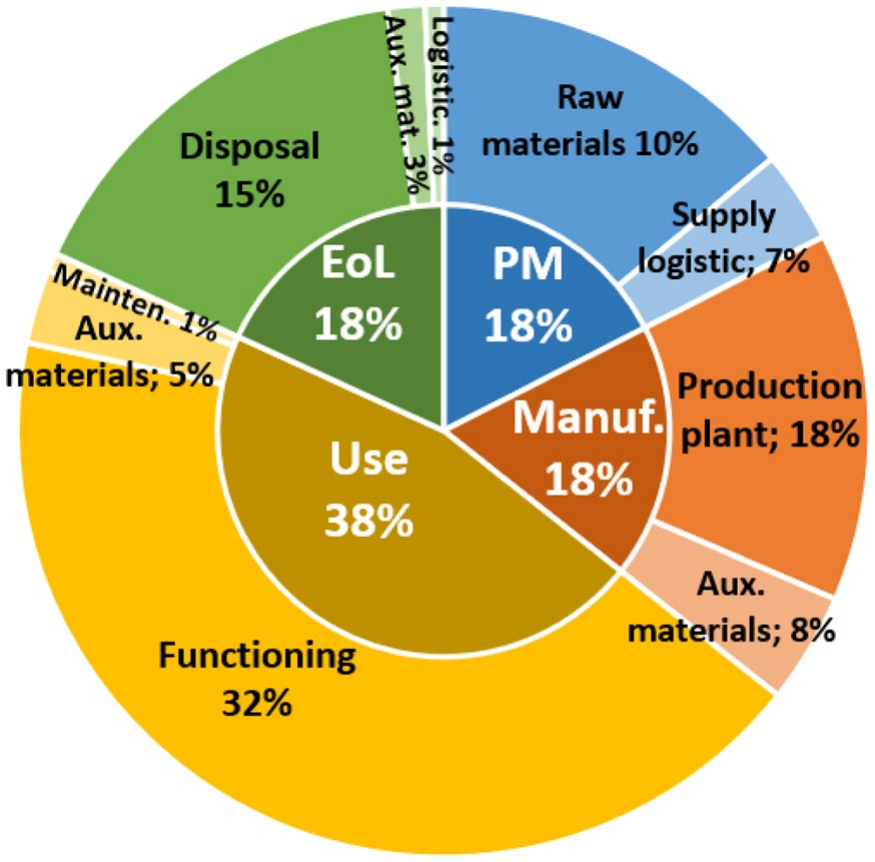
Classification of the failures identified by the considered methods according to the addressed items and product life cycle phases (where “Manuf.” = Manufacturing)

Figure 2 graphically summarizes the obtained results.

Analysing the results shown in Table 1 and Fig. 2, we note first the interest of the considered methods towards the identification of failures related to the use phase of the product, present in almost 40% of the documents, followed at a distance by those investigating the failures of manufacturing.

As regards the distributions within the single phases, we apprehended that, in pre-manufacturing, there is a much greater interest in the failures relating to raw materials; in manufacturing, the main focus is on the production plant; during use, the identified failures mainly concern the product functioning rather than the user’s misuses; and finally, in the end-of-life, the focus is on the problems occurring during disposal, and their resulting environmental contamination.

### Exploited approaches

In this section, we present the approaches exploited by the considered methods to determine the environmental failures, as explicitly stated in the related documents.

All the identified approaches have been grouped into five classes: (1) *FMEA-based* are structured methods that allow the identification of hypothetical failures of all the components of a product, through a rigorous procedure divided into steps to be sequentially followed. (2) The *tests* are used to deliberately bring out the failures in the product or in a sample of it, appropriately stressing its behaviour or varying the ad hoc environmental conditions. (3) *Historical failure databases* are a source of knowledge relating to possible known failures of a product equal or similar to the one analysed. (4) *Simulations* are a virtual alternative to physical tests, typically used to simulate a greater number of conditions for the onset of risks and save execution time and costs, and (5) *requirement perturbation* and analysis of their consequences.

**Fig. 3:**
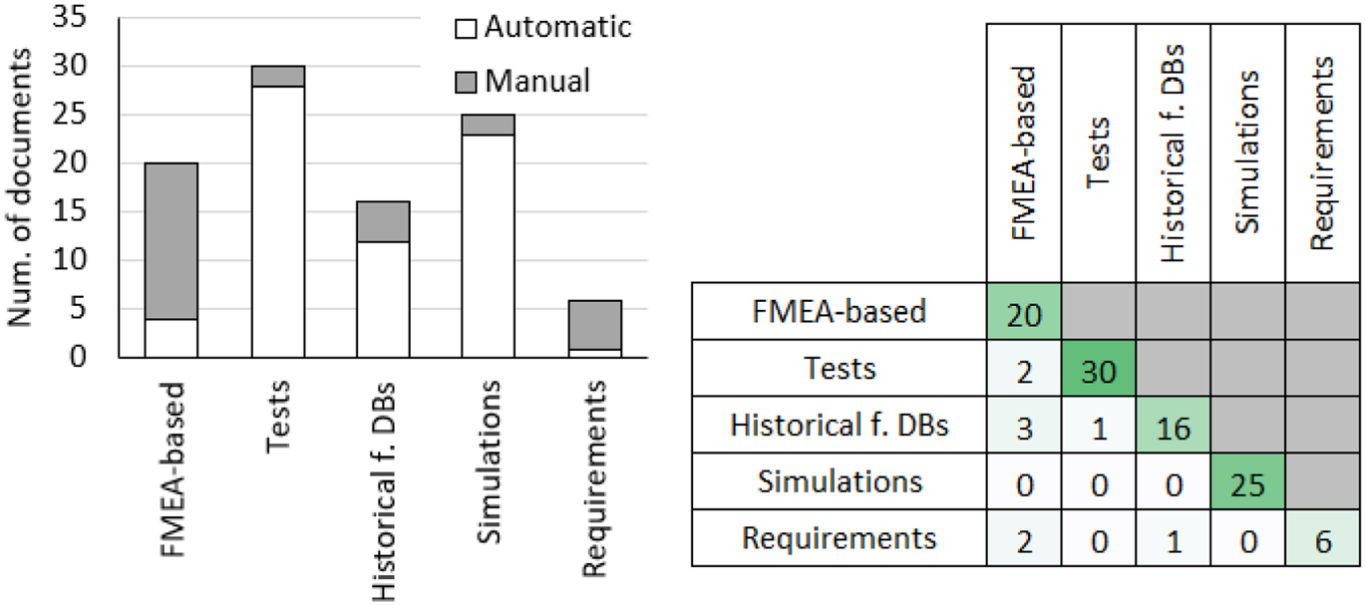
(left) Distributions of the failure determination approaches and (right) number of intersections between them within the considered documents

Figure 3 (left) shows the comparison between the numbers of documents referring to each failure determination approach, dividing them among those that allow the automatic and manual identification of failures, while Fig. 1 (right) reports the number of intersections between the different approaches within the analysed documents.

Analysing Fig. 3, we note first that tests, simulations and FMEA-based methods are the most exploited approaches to determine the failures. Considering instead the repercussions that these approaches have on failure determination activity, we learn that those allowing to automate the search for failures are many more of those involving manual search (68 vs 21). In particular, the latter is exploited predominantly only in FMEA-based approaches and in those based on the perturbation of requirements. Finally, analysing the intersections between the methods, we note that there are some attempts to combine FMEA-based approaches with other approaches to determine the failures.

To better explain how the identified approaches work, in Table 3, they were compared with the top 5 application fields (presented in Fig. 1) and with the classes of the types of failures (see “Types of failure”), based on the number of documents identified for each intersection.

**Table 3:**
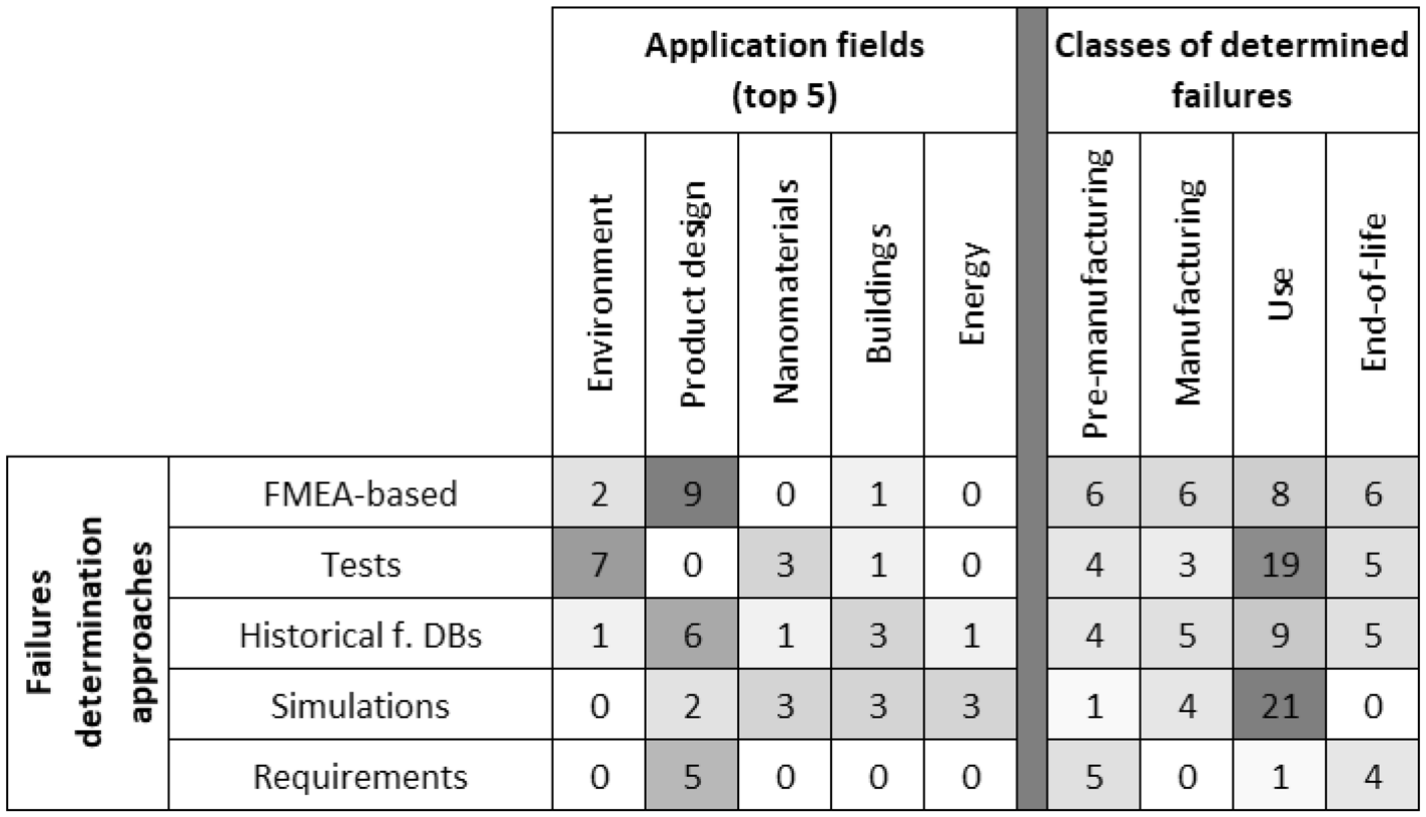
Intersections of the number of documents between failure determination approaches, application fields and classes of failures

Analysing Table 3, as regards the application fields, it can be seen that FMEA-based approaches are used to determine failures especially for product design, along with historical failure databases, and tests are preferred for the failures of the environment and waste management. While, in the case of the classes of the determined failures, those of the use phase are almost equally investigated, especially by using tests and simulations; FMEA-based are instead preferred for determining the failures of the other phases. Finally, simulations are used almost exclusively for the failure during the use.

In the following paragraphs, the main identified results for each failure determination approach are presented in detail.

#### FMEA-based

FMEA-based methods best known are E-FMEA (Nilsson et al., 1998) and Eco-FMEA (Dannheim et al., 1998), which have modified and adapted the traditional FMEA with the aim of making it an eco-design tool and almost all other FMEA-based approaches claim to be based on them. A distinctive feature of all these methods is the introduction of an ontology more oriented towards eco-design as well as a new metric for risk assessment, with the common focus that was on increasing the effectiveness of determining a greater number of failures, especially with net repercussions on the environment. There have been no particular efforts to improve the applicability, reducing time and operating costs, as was done for many FMEA improvements in other application fields (Spreafico et al., 2017). In addition, some of them have aimed at improving the pragmatism of the approach, with the introduction of new supporting methods, such as the root cause analysis (Djatna & Prasetyo, 2019) which has proved particularly suitable for identifying multiple failures linked to each other by complex cause and effect chains, while those combining FMEA with tests (Yazdani et al., 2019) and historical failure databases (Lewandowska et al., 2017) always maintain its basic and, in most cases, manual supervision scheme but were able to increase the reliability of the analysis being able to confirm the hypothesized failures.

#### Tests

The identified tests, used to detect the failures, can be classified into two types: on the one hand, there are stress tests that aim to deliberately provoke the failures in the product, subjecting the latter to particular stresses or environmental conditions (e.g. Kishore & Adhikary, 2019); while on the other hand, failures are detected only when they naturally occur, by monitoring the product during part of its life, but without interfering in any way (e.g. Ahn et al., 2019). Stress tests are performed in laboratories in most cases, often by experimenting samples rather than the entire product, and they can reduce the execution times, explore many different conditions, and increase the number of identified failures. On the other hand, particular attention must be paid to the reliability of the tests in relation to their representativeness with respect to the real conditions. The direct detections of the failures have been used, for example, to measure the emissions of contaminants into the air or water because of perforations and gaps in containment structures. Also, in this case, the detection procedure is automatic, although it is however much more expensive in terms of time than the previous one.

#### Historical failure database

The use of historical failure databases has the advantage of significantly reducing the time for detecting failures, compared to other structured and mainly manual approaches such as FMEA (Lazarova et al., 2012). Their use is especially suitable when performing the eco-assessment on a widespread product or that includes components whose failures are known or easily retrievable by similar components, reported in the databases. Fortunately, over the years, the amount of information available and the cases treated by the latter has significantly increased, and consequently, the representativeness of the retrievable failures in relation to the analysed product, by making this tool an increasingly valuable option (Favi et al., 2018). In addition, in some cases, the use of such databases also allows the complete automation of the failure determination phase, by implementing the databases within software-based approaches, that able to perform also risk analysis and eco-assessment (e.g. Hennequin et al., 2018).

#### Simulations

Among the documents considered, the virtual simulations most used are those based on the finite element (FEM) method (e.g. Singh & Abdullah, 2019), which are almost exclusively related to the detection of structural failures. They can also be implemented within software, providing the results directly and automatically to the risk analysis and eco-assessment modules.

In other cases, the failure simulations have been developed ad hoc to study the negative effects of different operational scenarios, specifically modifying the boundary conditions (of the simulation) with respect to the parameters defined within the functional unit of the eco-assessment, and they are also the preferred approach, among those analysed, to investigate failure deriving from user’s misusing during the product functioning (e.g. Norazahar et al., 2018). In all the analysed cases, simulations have the advantage of being able to apply the anticipatory failure investigation in eco-design, allowing the estimation of the impacts of a product at the design or prototype stage but which has not yet been manufactured and marketed. Furthermore, in this context, compared to other more manual approaches such as Design-FMEA, simulations can characterize failures in a considerably more complete, precise and reliable way, although mostly limited to structural failures during operation or manufacturing (Naderi et al., 2020).

#### Requirement perturbation

The perturbation of requirements is a widely used approach to streamline the execution of the traditional FMEA (Spreafico et al., 2017) and also in the context of eco-assessment, among the analysed methods, the contributions that use this type of approach aiming to fulfil the same purpose, as well as improve the identification of failures (e.g. Lewandowska et al., 2017). Furthermore, to facilitate the collection, management, and classification of the requirements to be disturbed, especially when they are many, integration with dedicated approaches has also been proposed such as the quality function deployment (QFD) method was proposed by Sakao et al. (2008).

## Risk assessment approaches

In this section, we present the approaches exploited by the considered methods to assess the environmental risks, as explicitly stated in the related documents.

**Fig. 4:**
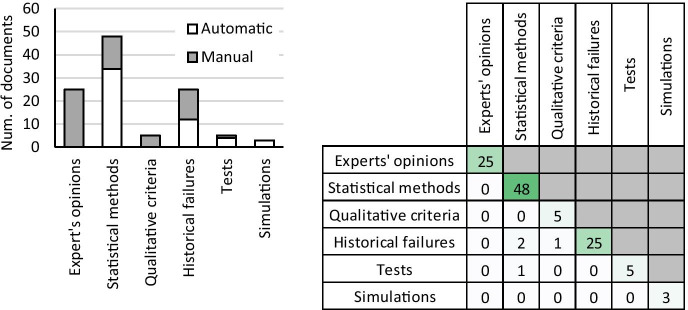
left) Distribution of risk analysis approaches and (right) numbers of their intersections within the analysed documents

All the identified approaches have been grouped into six classes: (1) *experts’ opinions*, i.e. subjective assessments based on experience; (2) *statistical methods* providing probabilistic risk estimated through a certain confidence interval; (3) *qualitative criteria* based on generic and un-domain textual explanations; (4) *historical failure databases* containing risk values already calculated in cases similar to the analysed one; (5) *tests* to obtain the exact value of the risk, directly evaluating the probability of occurrence of real failures; and (6) *simulations* that represent the virtual analogue of the tests (Fig. 4).

Figure 4 (left) shows the comparison between the numbers of documents referring to each risk assessment approach, dividing them between those allowing automatic and manual analysis, while Fig. 1 (right) reports the number of intersections between the different approaches in the considered pool of documents.

Analysing Fig. 4, we apprehend that statistical approaches are widely used for risk assessment, followed by experts’ opinions and historical failures, while the other approaches count significantly less contributions. In this case, differently from failure determination, the manual methods (58 documents) are more than the automatic ones (53 documents), where the statistical approaches are the most suitable for automation. Finally, from the analysis of the intersections between the risk assessment approaches, we note that almost all the considered documents focus exclusively on the implementation of a single method.

To better explain how the main risk assessment approaches work, in Table 3, they have been compared with the top 5 application fields (presented in Fig. 1) and with the classes of the types of failures (see “Types of failure”), based on the number of documents identified for each intersection.

Analysing Table 4, as regards the application fields, we can see that statistical methods are mainly used to analyse the risks about environment and product design, while historical databases are mainly exploited for those about environment. For what concerns the classes of determined failures, the use of statistical methods is associated overall with the failures during the product use phase (Table 5).

**Table 4:**
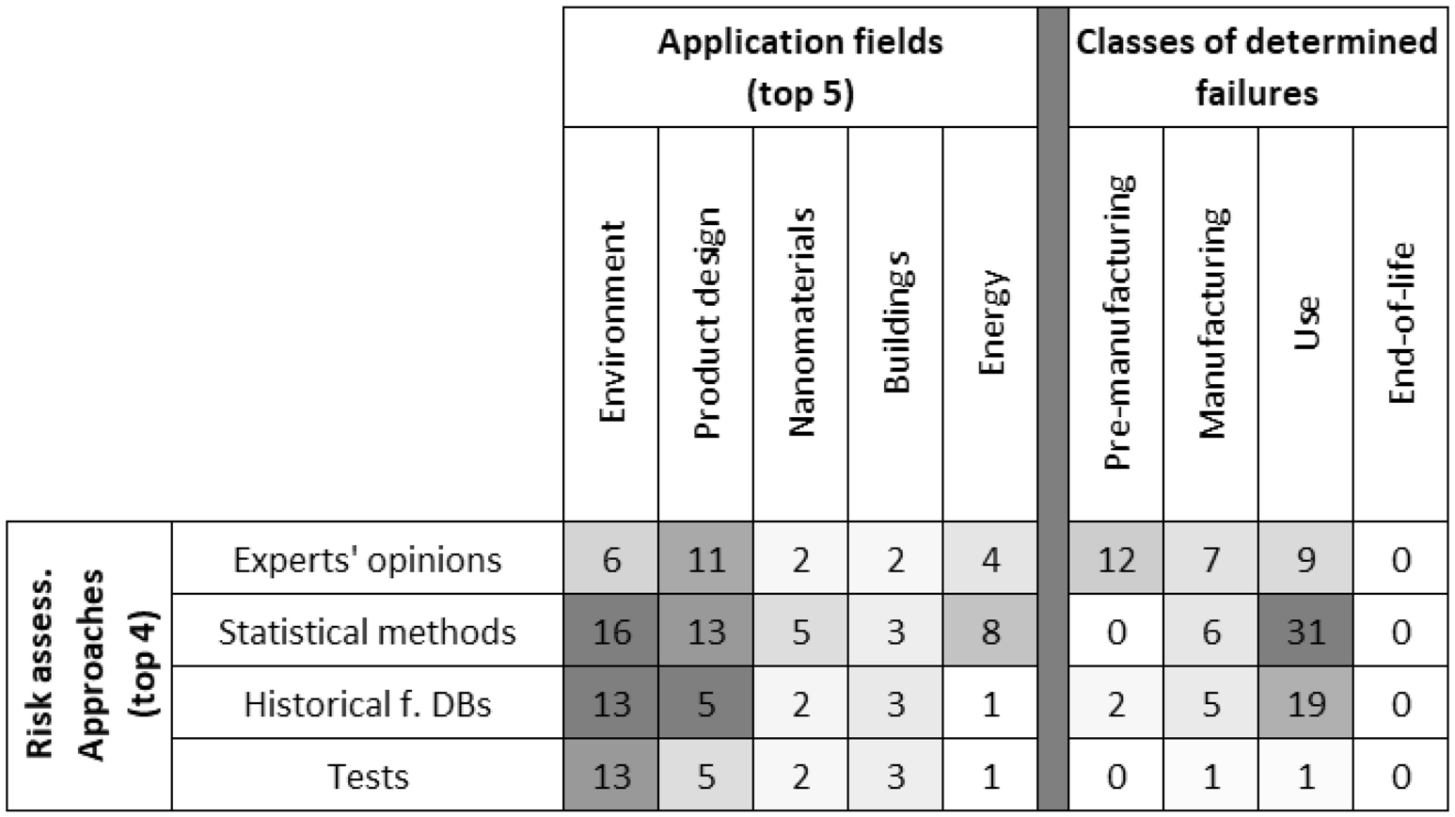
Intersections of the number of documents between risk assessment approaches (top 3), application fields and classes of failures

In the following paragraphs, the main identified approaches for risk analysis are presented in detail, explaining their main advantages and disadvantages in the context of eco-assessment.

### Experts’ opinions and qualitative criteria

In these approaches, the experts’ opinions are generally used within a structured risk calculation methodology, based on personal and non-automatable responses. Among them, the most used is FMEA (e.g. Djatna & Prasetyo, 2019) exploiting the risk probability number (RPN) to assess the risk, which is the product of three factors: probability of occurrence, severity of the effect associated with the failure and possibility of detecting the failure. These factors are estimated independently by the experts, based on a scoring system, supported by some general indications explaining the meaning of the values associated with them.

Some approaches, starting with Nilsson et al. (1998) have replaced the general metric with a more specific and specifically defined one to recall the typical jargon of eco-assessment, which refers to its problems and its most widespread issues. Consequently, by increasing the awareness about the problem, the main advantage of these approaches concerns the reduction of the subjectivity of the provided judgments. Finally, to retrieve a greater number of assessments, shared platforms have also been introduced (e.g. van Harmelen, 2016) which allow interfacing with experts who work remotely and to store and share their opinions.

The use of qualitative criteria, on the other hand, consists in facilitating the risk analysis by the experts, providing them with (qualitative) information relating to the environmental problems of certain failures that replace the generic criteria used to estimate the factors constituting the RPN in the FMEA, in both the identified approaches of this category (Kozhovska, 2016; De Almeida and Castro, 2015).

### Statistical methods

Different types of statistical methods have been identified to analyse the risk of failures for eco-assessment, each of them with its own peculiarities.

Qualitative prediction models use statistical tests to assess risk in a mainly automatic way, such as the Decision-Making Trial and Evaluation Laboratory (DEMATEL) method (Yazdani et al., 2019) or the Monte Carlo method (Hennequin et al., 2018), or expert team management methods, for more manual evaluation, such as the Delphi method (Djekic et al., 2019), to guide evaluators through a statistical analysis during decision-making. In the analysed cases, these approaches are combined with very structured procedures of both failure determination (FMEA) and risk assessment (LCA), mainly due to their pragmatism and precision (Hennequin et al., 2018). On the other hand, their implementation is rather laborious when compared with other alternative approaches (Djekic et al., 2019).

The Markov chain model (e.g. Singh & Abdullah, 2019) is used to determine the risk of failures, typically arising from single events whose relations are complex and non-deterministic, such as in the case of structural failures in assemblies with many components, reciprocal movements and energy flows of various kinds.

The statistical functions allow to estimate the risks of failures by associating them with a calculation model if compatible. For instance, Norazahar et al. (2018) to estimate the possibility of a failure occurring in an offshore environment, they recovered the probabilistic function from two other previously analysed publications having remarkable similarities with the considered applicative case. In this case, they provide risk estimates with rather high reliability coefficients and with repeatable results.

The cumulative damage function (e.g. Deo et al., 2017) is used to predict the possibility of a failure occurring as a function of the progress of some characteristic parameters (e.g. crack propagation in a fatigue failure). For this reason, its use is typically associated with products working in a damage tolerant regime (e.g. aeronautical components), and the risk assessment, with this approach, can also be estimated automatically and online during the functioning of the product.

Finally, the regression model (e.g. Gernes et al., 2019) allows to link together different influencing factors obtained from tests and otherwise difficult to correlate with each other to analyse risks.

### Historical failure databases

Retrieving risk values from databases can guarantee advantages both at the applicative level, by reducing time and cost of this activity, and on the reliability of the result, if cases similar to the one analysed can be identified in the literature (Palit et al., 2019). In addition, the ever-increasing expansion of risk databases, albeit with reference to standard components in most cases, pushes to rely more and more on this approach.

In some cases, the data recovered from the historical databases are also used within other statistical-probabilistic approaches (e.g. Pettersen & Hertwich, 2008), mainly in order to speed them up, exploiting the available data in most cases and delegating the calculation with the precise analysis only to analyse the non-standard failures.

### Tests and simulations

In all considered cases, tests were used to analyse failure risks that are difficult to model with other approaches, and they are performed directly on the product during its functioning. For example, Treumann et al. (2014) tested the anomalies of a chemical colloid adsorption process, noting that the results obtained from column (real functioning) and sample experiments were generally not comparable, and predictions based to the classical reference theory were found to inadequately describe the failures within the colloid.

Virtual simulations instead propose the use of mathematical models to determine the risk and are often associated with the eco-assessment of nanomaterials (e.g. Cullen et al., 2010). The major problems concern the reliability and significance of the result obtained in relation to the boundary conditions that are set and the compatibility of the model with the studied problem.

## Eco-assessment approaches

In this section, we present the approaches exploited by the considered methods to assess the environmental impacts, related to the identified failures and in relation to their risks of occurrence, as explicitly stated in the related documents.

Among them, three approaches were identified: (1) *determination of direct pollutants* by experimental detection of fluid and solid emissions of materials harmful to environment and humans, and measurement of their exact quantities (e.g. Ahn et al., 2019). (2) *Generic assessment*, providing an assessment of environmental impacts according to the most well-known indicators, but without following a rigorous and regulated procedure (e.g. Norazahar et al., 2018). (3) *LCA* methodology application according to the strict application of the reference standards (e.g. ISO 14040: 2006 (ISO, 2006a) and ISO 14044: 2006 (ISO, 2006b)) in relation to the assumptions and calculation procedures, in order to identify the indicators environmental impact standards (e.g. Kishore & Adhikary, 2019).

**Fig. 5:**
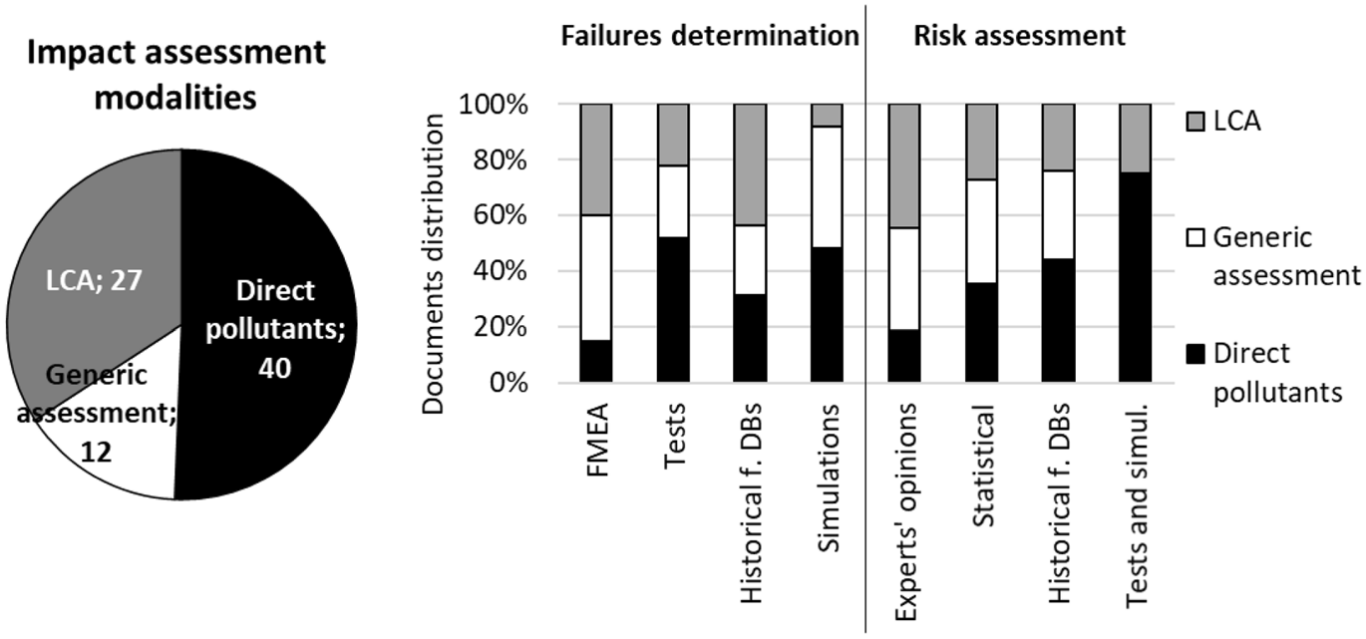
(left) Distribution of impact assessment modalities in the considered methods and (right) comparison with failure determination approaches and risk assessment approaches

Figure 5 (left) presents the distribution of the impact assessment modalities according to the considered documents and (right) compares their intersections with the failure determination approaches and the risk assessment approaches, according to the percentage distributions of the number of related documents.

Analysing Fig. 5 (left), we can apprehend that, among the impact assessment modalities, the determination of direct pollutants is the most considered, in over half of the cases, followed by LCA.

From the comparison between the eco-assessment modalities and the failure determination approaches (see Fig. 5 — right), we can learn that there is no strong link between them and one of the eco-design approaches. The use of LCA is the most variable: simulations practically do not consider it, while FMEA and historical features database do. On the other hand, in the case of the intersections between the eco-assessment modalities and the risk analysis approaches, there are most evident links between test and simulations with the determination of the direct pollutants.

**Fig. 6:**
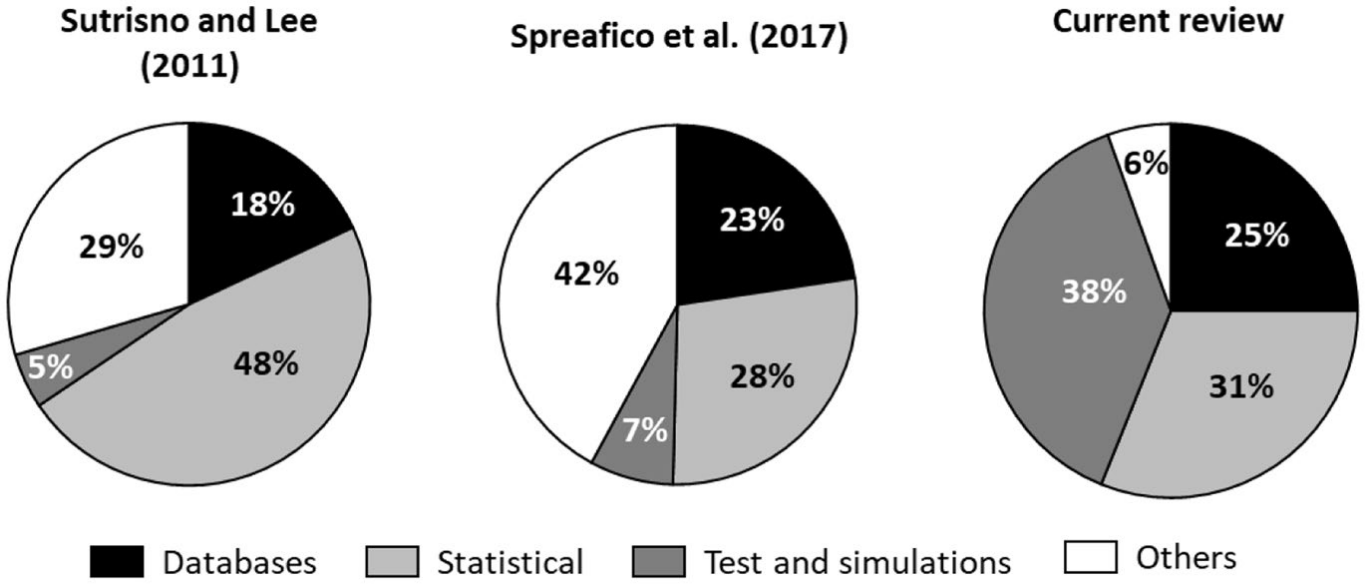
Classification of the methods and tool in the reviews of Sutrisno and Lee (2011), Spreafico et al. (2017) and the current one

Analysing the considered methods in more detail, we can comprehend in what ways and for what purposes the identified eco-assessment approaches are combined with failure determination and risk assessment approaches.

For what concern the intersections of LCA and FMEA, we identified three main approaches. FMEA combined with the statistical risk estimate allows us to significantly improve the definition of the functional unit in the LCA, replacing the assumptions made a priori, with updated values even during use. In this context, Deo et al. (2017) proposed an iterative calculation method of the LCA that updates the residual life of the product, depending on the failures that gradually occur during use, by introducing a cumulative damage function. Another possibility is to use FMEA to make the LCA inventory more scientific, by determining the quantities of the considered materials in a statistical way according to the risks of their failures, also considering the spare parts (e.g. Djekic et al., 2019). Still another is to apply FMEA exclusively as anticipatory failure investigation methodology to determine the possible environmental failures to be avoided through eco-improvement, where LCA is carried out both before and after this activity for evaluating its efficacy (Djatna & Prasetyo, 2019).

Statistical methods to analyse risk can be the only way to solve some typical errors in LCA, deriving from deterministic estimates or inaccuracies in data collection based on experiments. This can occur when exploring the environmental impacts related to emissions of polluting materials that are difficult to detect, such as nanomaterials or fine dust (e.g. Ettrup et al., 2017). Furthermore, statistical correlations allow to order a set of data obtained from tests in complex scenarios, where it is not possible to extrapolate exact data be used within LCA (e.g. Pivato et al., 2016).

Finally, the identified methods mainly exploit experts’ opinions for risk analysis for LCA to confirm statistical evaluations or tests (e.g. Lazarova et al., 2012) or to replace them if the intervals of confidence are not satisfactory, models from literature are not available or tests are not very significant, as in innovative products, still at the design or prototype stage (Djatna & Prasetyo, 2019).

## Discussion of the results

This section presents a critical discussion of the outcomes, with the aim of comparing the methods for the evaluation of failures in the eco-assessment, analysed in this study, with the traditional approaches of failure investigation (“Comparison with traditional failure investigation approaches”) and eco-assessment (“Comparison with traditional LCA”).

### Comparison with traditional failure investigation approaches

To compare our results with those about the traditional failures investigation and risk analysis approaches, we considered the reviews of Sutrisno and Lee (2011) and Spreafico et al. (2017), which provide a very broad overview on the subject, with a particular focus on FMEA, analysing, respectively, 65 scientific papers and 329 documents, of which 220 scientific papers and 109 patents, published from the late seventies to 2017.

To obtain a first general comparison, all the methods and tools analysed in the two reviews were grouped using the same classes described in this study (see “Exploited approaches” and “Risk assessment approaches”), without discriminating their use for failure determination or for risk analysis. In this comparison, we excluded FMEA-based approaches, since they are present in almost all cases in previous reviews and experts’ opinions, which cannot be considered among the supporting tools.

The comparison between the three obtained classifications is shown in Fig. 6.

From the analysis of Fig. 6, there is a clear difference between this review and the others, especially regarding the extensive use of tests and simulations, recalling that the first ones are significantly greater in number than the second ones (see Fig. 3). This increase is mainly to the detriment of the use of “others”, namely, infographics, ontologies, scenario analysis, project management models, decision-making and team management methods, and requirement management tools. The use of statistical methods, albeit variable, accounts for a good percentage of the total in all three cases, while the use of historical failure databases is comparable in all studies.

**Fig. 7:**
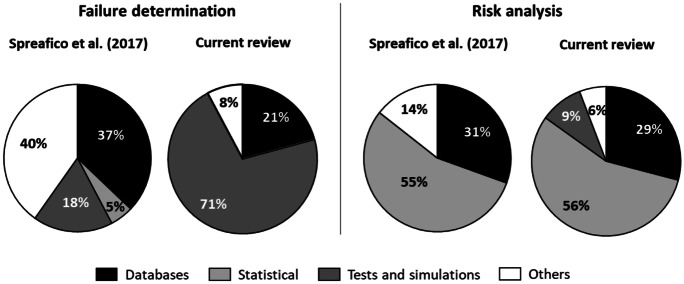
Classifications of methods and tools for failure determination and risk assessment in Spreafico et al. (2017) and in this review

Going into more detail of the comparison, we were also able to discriminate the methods and tools of the various classes according to the two phases of failure determination and risk analysis (see Fig. 7), although in this case, we can compare our study only with the review by Spreafico et al. (2017) which deepening is compatible with this level of detail.

By analysing Fig. 7, we can learn the usefulness of the stratification proposed in the discussion of the results. In fact, regarding the context of failure determination, we can see a much greater use of tests and simulations (+ 53%) in this study, especially to the detriment of others (− 32%). Therefore, this fact highlights the importance of obtaining experimental evidence when detecting environmental failures. Historical failure databases seem slightly less used in our case, and statistical methods are not even contemplated. Instead, for what concerns risk analysis, the greatest discrepancies arise in the use of tests; unused in the general case, which practically replace others; and in the almost halved use of historical failure databases.

### Comparison with traditional LCA

**Fig. 8:**
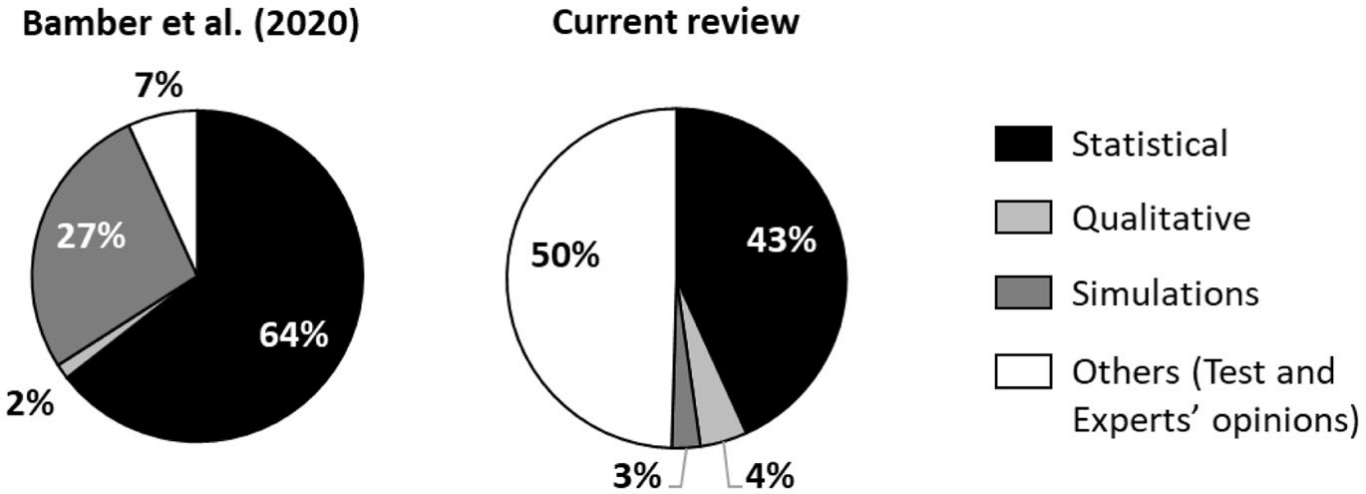
Classification of the methods and tool in Bamber et al. (2020) and in our review

In order to compare our results with those of the traditional LCA, we used the review by Bamber et al. (2020) as feedback. It compares and classifies 2687 studies combining attributional and consequential LCA, published in international scientific journals in the sector between 2014 and 2018, with the aim of providing a general and complete overview on the subject. Also, in this case, as in “Comparison with traditional failure investigation approaches”, we compared the classifications of the exploited methods and tools (see Fig. 8), albeit partially modifying the contents of the classes.

Analysing Fig. 8, some peculiarities of the considered methods can be highlighted in comparison with the generic eco-assessment approaches, which can be summarized mainly in a much greater expense towards tests and experts’ opinions, in the drastic decrease in the use of simulations and in the reduction of statistical methods, while the use of qualitative criteria is limited in both the reviews.

Going into more detail of the individual methods, we also found considerable reductions in the application of the most popular traditional LCA tools such as the Monte Carlo method, which is generally implemented in most of the commercial software. It accounted for 61% of overall cases in the review by Bamber et al. (2020), while in our study, it is not even the most widespread among statistical approaches. Another difference concerns the sensitivity analyses, which according to Bamber et al. (2020) are used in 19% of cases, while in the methods considered by our study, they are almost ignored.

## Conclusions

This paper proposes a review about the methods for identifying failures and assessing their risks during eco-assessment. The basis of evaluation is constituted by a set of 106 scientific documents, each proposing a single method, while the main approximation of the study concerns the manual analysis and classification of the considered sources. From each, analysed method has been extracted: the application field; the type of failures that it can determine in relation to product life cycles items; the exploited failure determination approaches; the risk analysis approaches, for evaluating the risks connected to the determined failures; and the eco-assessment approaches, for calculating the environmental impacts arising from the determined failures and depending by their risks of occurrences. The main outputs of this work have been summarized in some explanatory graphs representing different classifications of all the analysed items, tables for identifying the intersections between the different classifications and a detailed discussion of the advantages and disadvantages, in relation to the topic of eco-assessment, of all the main identified approaches.

In conclusions, the proposed study provided the elements to answer the research questions.

RQ1: The analysis of the types of failures identified by the considered methods showed great attention to those relating to the product functioning, especially for the identification of mechanical failures and malfunctions, followed by the other phases, while analysing the failure determination approaches, a greater consideration of tests and simulations, especially for those of the use phase.

RQ 2: To analyse the risk, the statistical methods are the most widely used approaches, followed by the experts’ opinions and historical failures, where the first ones are particularly suitable to objectify and automatize this procedure, and they are also particularly effective for assessing the risks associated with failures occurring during the product functioning.

RQ 3: Analyzing the methods for assessing the environmental impacts associated with failures, a clear interest in determing the direct pollutants emerged. In addition, these latter are generally combined with tests and simulations to quantify the pollutants related to the potential failures.

Finally, when discussing the obtained results by comparing them with the previous general reviews on failure risk analysis and eco-assessment, some differences emerged for what concerns the exploited approaches, in particular as regards the greater use of tests to substitute more structured approaches, such as methodologies for requirement management and simulations.

By virtue of the achieved considerations and in relation to the limitations of our work, we retain that this study has highlighted a growing interest on this argument within the scientific community, which was expressed in many different application fields of the proposed methods and the journals that published them. Furthermore, this discipline has so far been approached in a rather different and original way compared to traditional approaches of both failure risk analysis and eco-assessment. For these reasons, if the industry will also be more interested in this argument, then it will have to focus on the development of new skills: in this sense, the overview provided by this study could represent a first indicative framework for practitioners, suggesting the more appropriate approaches in relation to the application field and the type of environmental problems to be investigated.

## Appendix

**Table 5:** Main classification of the analysed documents according to the four criteria of investigation (where PM = pre-manufacturing, M = manufacturing, U = use, EoL = end-of-life; DP = direct pollutants; GE = generic evaluation; LCA = life cycle assessment)

Author	What failure	Failure determination approaches	Risk assessment approaches	Eco-assessment approaches
Ahn et al. (2019)	Tests	U	Statistical	DP
Alsaqqaf and Zhang (2011)	Simulations	U	Statistical	GE
Anand and Wani (2010)	Experts	PM, U, EoL	Experts	GE
Antunes et al. (2014)	Tests	EoL	Tests	DP
Aoyagi and Ogunseitan (2015)	Simulations	M	Statistical	DP
Arlitt et al. (2017)	Historical f. DBs	PM, U, EoL	Historical f. DBs	LCA
Ashbolt (2015)	Tests	U	Statistical	DP
Aygün and Baycan (2020)	Experts	PM, U, EoL	Statistical	GE
Bai et al. (2017)	FMEA	PM, U, EoL	Statistical	GE
Boboc et al. (2014)	Experts	PM, U, EoL	Statistical	GE
Bradley et al. (2020)	Simulations	M	Statistical	DP
Breedveld (2013)	n.a	U, EoL	Experts	LCA
Brown (2012)	Experts	U	Experts	GE
Cedola and Stasio (2001)	Experts interrogation	PM, U, EoL	Experts	LCA
Cullen et al. (2010)	Simulations	U	Simulations	DP
Dannheim et al. (1998)	FMEA	U	Experts	GE
Dargahi et al. (2016)	FMEA	U	Experts	DP
De Almeida Júnior and Castro (2015)	FMEA	U	Qualitative	LCA
Deo et al. (2017)	FMEA	U	Statistical	LCA
Djatna and Prasetyo (2019)	FMEA	M	Experts	LCA
Djekic et al. (2019)	FMEA	PM	Statistical	LCA
Ebel and Davitashvili (2006)	Simulations	U	Statistical	GE
Ettrup et al. (2017)	Tests	U	Statistical	LCA, DP
Favi et al. (2018)	Historical f. DBs	M	Historical f. DBs	LCA
Fenner-Crisp and Dellarco (2016)	Historical f. DBs	U	Historical f. DBs	DP
Gernes et al. (2019)	Tests	U	Statistical	DP
Ghodous (2006)	Historical f. DBs	M	Experts	LCA
Gough et al. (2020)	Tests	U	Statistical	DP)
Guitart et al. (2013)	Simulations	U	Statistical	GE
Hennequin et al. (2018)	Historical f. DBs	PM, M, U	Statistical	LCA
Herman and Janasak (2011)	FMEA	PM, M, U	Experts	DP, GE
Huang et al. (2008)	Simulations	U	Historical f. DBs	GE
Huete and Didan (2004)	Tests	U	Experts	GE
Ibrahim and Chassapis (2017)	Statistical	M	Experts	GE
Jang et al. (2013)	Tests	PM	Statistical	DP
Joan et al. (2020)	Tests	U	Historical f. DBs	DP
Kang et al. (2005)	Requirements	PM, U, EoL	Experts	LCA
Keshavarzi and Kumar (2020)	Experts	U	Historical f. DBs	DP
Kiran et al. (2011)	Simulations	U	Statistical	GE
Kishore and Adhikary (2019)	Tests	PM, M	Experts	LCA
Koch et al. (2007)	Tests	U	Historical f. DBs	DP
Kozhovska (2016)	FMEA	M, U	Qualitative, historical f. DBs	LCA
Kulińska and Giera (2020)	Experts	PM, U, EoL	Qualitative	GE
Lazarova et al. (2012)	FMEA, tests	EoL	Tests, experts, statistical	LCA
Leichang et al. (2009)	Simulations	M	Historical f. DBs	GE
Lemos and Castro (2018)	FMEA	EoL	Experts	LCA
Lewandowska et al. (2017)	FMEA, Requirements	PM, EoL	Experts	LCA
Li et al. (2011)	Historical f. DBs	U	Statistical	DP
Li et al. (2019)	Tests	U	Experts	DP
Lindahl (1999)	FMEA, historical f. DBs, requirements	M, U	Historical f. DBs	GE
Liu et al. (2018a, b)	Simulations	U	Statistical	DP
Liu et al. (2018a, b)	Simulations	U	Statistical	DP
Liu et al. (2020)	Experts	U	Historical f. DBs	DP
Masood and Java (2015)	Tests	U	Historical f. DBs	GE
Matthews et al. (2002)	n.a	M	Statistical	LCA, DP
Moskwa and Alby (1999)	Historical f. DBs	EoL	Historical f. DBs	DP
Mouron et al. (2006)	Tests	M	Statistical	LCA
Naderi et al. (2020)	Simulations, Statistical	U	Statistical	LCA
Newman et al. (2000)	Experts	U, EoL	Statistical	LCA, DP
Ngole-Jeme and Fantke (2017)	Experts	M	Historical failure analysis	GE
Niemeyer et al. (2015)	Simulation (mathematical model)	U	Statistic (probabilistic)	DP
Nilsson et al. (1998)	FMEA, historical f. DBs	M, U, EoL	Historical f. DBs	GE
Norazahar et al. (2018)	Simulations	U	Statistical	GE
Oka et al. (2014)	Tests	U	Tests	DP
Olsen et al. (2016)	Experts	PM, U, EoL	Statistical	GE
Ono et al. (2019)	Experts	U	Statistical	GE
Owens (1997)	Experts	U	Statistical	LCA
Palekhova et al. (2015)	Requirements	PM, U, EoL	Experts	LCA
Palit et al. (2019)	Historical f. DBs	PM, U	Historical f. DBs	LCA
Peck and Bakker (2012)	Requirements	PM, U, EoL	Experts	LCA
Pettersen and Hertwich (2008)	Historical f. DBs	U	Statistical, historical f. DBs	LCA
Pivato et al. (2016)	Tests	U	Statistical	LCA
Rajkovich and Okour (2019)	Experts	M	Experts	GE
Rochlin et al. (2011)	n.a	U	Statistical	DP
Roszak et al. (2015)	FMEA	M	Experts	DP, GE
Sakao et al. (2008)	Requirements	PM	Experts	LCA
Savoskula et al. (2019)	Tests	PM, U	Experts	LCA
Schnetgöke et al. (2015)	Historical f. DBs	U	Statistical, historical f. DBs	LCA
Shi (2010)	Tests	U	Experts	DP
Shilin et al. (2018)	Simulations	U	Historical f. DBs	DP
Singh and Abdullah (2019)	Simulations	U	Statistical	GE
Sousa et al. (2020)	Simulations	M	Statistical	DP
Su et al. (2018)	Tests	EoL	Experts	GE
Sun et al. (2020)	Experts	U	Statistical	GE
Tian et al. (2019)	Simulations	U	Historical f. DBs	DP
Tian et al. (2021)	Simulations	U	Historical f. DBs	DP
Tingley and Shine (2011)	Simulations	U	Statistical	GE
Treumann et al. (2014)	Tests	M	Tests	DP
van Harmelen (2016)	Experts	PM	Experts	GE
Vazdani et al. (2017)	FMEA, historical f. DBs	M	Simulations	GE
Wang (2021)	Tests	U	Historical f. DBs	GE
Wang and Tian (2012)	Simulations	U	Statistical	DP
Wang et al. (2012)	Tests	U	Historical f. DBs	DP
Wang et al. (2016)	Tests, historical f. DBs	U, EoL	Statistical	DP
Wang et al. (2019)	Tests	EoL	Statistical	GE
Wen et al. (2009)	Simulations	U	Historical f. DBs	GE
Withanage et al. (2016)	Tests	U	Statistical	GE
Yazdani et al. (2019)	FMEA, tests	PM	Statistical	GE
Yen and Chen (2005)	Historical f. DBs	PM, U, EoL	Experts	GE
Yen and Chen (2005)	FMEA	PM, U, EoL	Statistical	GE
Yoon et al. (2018)	Simulations	PM, U	Simulations	LCA
Yuan et al. (2020)	Experts	U	Qualitative	DP
Zeimes et al. (2014)	Simulations	U	Statistical	GE
Zheng and Sun (2011)	Tests	U	Historical f. DBs	DP
Zeng et al. (2010)	Historical f. DBs	U	Qualitative	DP
Zhou et al. (2018)	Simulations	U	Statistical	DP
